# Lessons learned from immunoadsorption for hyperviscosity in IgM multiple myeloma—A case report

**DOI:** 10.1002/jca.21775

**Published:** 2020-03-06

**Authors:** Philipp Gauckler, Johannes Leierer, Florian Kocher, Clemens Feistritzer, Wolfgang Willenbacher, Eberhard Gunsilius, Dominik Wolf, Hannes Neuwirt, Gert Mayer, Andreas Kronbichler

**Affiliations:** ^1^ Department of Internal Medicine IV (Nephrology and Hypertension) Medical University Innsbruck Innsbruck Austria; ^2^ Department of Internal Medicine V (Hematology and Oncology) Medical University Innsbruck Innsbruck Austria; ^3^ Oncotyrol, Center for Personalized Cancer Medicine Innsbruck Austria

**Keywords:** drug levels, immunoglobulin, monoclonal antibody, pembrolizumab, therapeutic apheresis

## Abstract

We report the case of a 63‐year‐old Caucasian woman with multiple relapsed IgM multiple myeloma (MM) and elevated free kappa light chains (fκLC). Due to hyperviscosity syndrome with visual impairment, regular plasma exchanges were performed. As part of her 11th line of therapy, an experimental protocol consisting of pembrolizumab, pomalidomide, and dexamethasone was initiated. To reduce fκLC and immunoglobulin (Ig) M, we performed immunoadsorption (IA) using columns containing recombinant single domain camelid antibody fragments as ligands. We measured pembrolizumab (humanized IgG4 kappa anti‐PD1 antibody) levels before and after each IA session and found a 98.1% reduction from baseline with five sessions of IA. Comparable elimination kinetics were observed for serum IgG, whereas fκLC and IgM were eliminated to a substantially lesser extent. These findings highlight that in hyperviscosity syndrome due to IgM MM, broad spectrum IA columns might be only moderately effective compared to total plasma exchange or double filtration plasmapheresis. Monoclonal antibodies are efficiently reduced by extracorporeal therapies and re‐dosing is necessary to provide sufficient efficacy. In the case of serious adverse events such as immune‐related adverse events, IA might be used to eliminate the monoclonal antibody. Measuring IgG levels might be a reasonable strategy for monitoring drug levels of monoclonal antibodies during IA.

## 1 INTRODUCTION

Multiple myeloma (MM) is a malignancy characterized by bone marrow infiltration with clonal plasma cells and production of monoclonal immunoglobulins and/or light chains. Treatment initiation is indicated when attributable end‐organ damage or myeloma defining events are present. Hyperviscosity syndrome due to high serum paraprotein levels is a rare major complication, most frequently seen in IgM‐related disorders like Waldenstroem's macroglobulinemia. Clinical symptoms such as mucosal bleeding, visual impairment and neurological symptoms warrant emergency therapeutic apheresis to quickly remove the causative paraproteins.^1^ While therapeutic plasma exchange (TPE) is the recommended apheresis modality, less evidence supports the use of immunoadsorption (IA) for hyperviscosity.^1^

Therapeutic apheresis eliminates concomitantly used biologicals. Only a few studies investigated the role of TPE to reduce toxic drugs.^2^ Immune checkpoint inhibitors, such as inhibitors of the protein programmed death 1 (PD‐1), reinvigorate an effective immune‐cell mediated anti‐tumor immunity.^3^ Pembrolizumab is a humanized monoclonal IgG4 kappa anti‐PD1 (149 kDA) antibody, approved for the treatment of various solid tumors. Except Hodgkin's lymphoma, experience with checkpoint inhibitors in hematological diseases is limited.^4^ Clearance occurs independently of metabolism with a terminal half‐life of 22 days.^5^

## 2 CASE REPORT

Here, we report the case of a 63‐year‐old Caucasian woman with MM evolving after a 20‐year history of IgM‐monoclonal gammopathy of undetermined significance. The rare diagnosis of IgM MM was established after exclusion of the prototypic Waldenstroem mutation of MYD88 L625P and the presence of myeloma bone disease.^6^

Prior therapies did not control her disease. Intermittent TPE was performed since 11 months because of hyperviscosity syndrome (initially presenting with retinal central vein occlusion with a total blood protein of 11.88 g/dL; for detailed information of the first two TPE sessions and technical details see Tables S1 and S4). Despite cycle II of 10th line therapy (cyclophosphamide, pomalidomide and dexamethasone) was just started, the patient presented with clinical signs of hyperviscosity syndrome. Total serum protein was 13.14 g/dL (6.70‐8.60) and IgM was 10.10 g/dL (0.041‐0.283) with an elevated kappa/lambda light chain ratio of 49.13 (1.35‐2.65). Thus, an experimental therapy protocol^7^ consisting of pembrolizumab (200 mg every 2 weeks), pomalidomide (3 mg on day 1 to 21; repeat on day 28) and dexamethasone (40 mg weekly) was initiated. In order to reduce plasma IgM and free kappa light chains (fκLC), five IA sessions using broad spectrum columns (for IA technical details see Table S3) were performed after the first application of pembrolizumab. To replace the eliminated drug after completed IA treatment, re‐dosing of pembrolizumab was administered at day 7. After the last IA session, total serum protein was reduced to 6.14 g/dL (53.3% reduction from baseline), IgM to 2.04 g/dL (79.8% reduction from baseline), and fκLC to 0.81 g/L (60.3% reduction from baseline). Further performance characteristics of IA are provided in Table 1. Specific protein elimination rates per session are listed in Table S2.

**Table 1:** Detailed overview of different parameters before and after each immunoadsorption session

IA session #	Pembrolizumab (serum) (μg/mL)			Albumin (3.56‐4.61 g/dL)		IgG (0.630‐1.610 g/dL)			Free light chain Kappa (0.0033‐0.0194 g/L)			IgM (0.041‐0.283 g/dL)			Complement C3 (90‐180 mg/dL)		Total protein (6.7‐8.6 g/dL)		Ionized calcium (mmol/L)		Total calcium (2.20‐2.55 mmol/L)	
	Pre	Post	Percent from baseline	Pre	Post	Pre	Post	Percent from baseline	Pre	Post	Percent from baseline	Pre	Post	Percent from baseline	Pre	Post	Pre	Post	Pre	Post	Pre	Post
I	51.04	11.99	23.5	2.04	1.93	0.21	0.06	28.6	2.04	1.36	66.7	9.5	7.0	73.7	85	69.9	11.45	9.18	1.22	1.34	2.27	3.22
II	14.76	3.15	6.2	2.28	2.04	0.1	0.02	9.5	1.98	1.15	56.4	7.2	4.6	48.4	89	65.6	10.17	7.44	1.06	1.09	2.64	3.30
III	4.69	1.36	2.7	2.28	1.94	0.05	0.01	4.8	2.06	1.05	51.5	5.9	3.6	37.9	81.8	70.2	8.94	6.44	1.21	1.13	2.34	2.02
IV	2.68	1.20	2.4	2.36	2.19	0.04	0.01	N/A	2.07	1.12	54.9	4.7	2.8	29.5	86.9	70.6	7.84	6.47	1.05	1.11	2.07	1.87
V	2.23	0.96	1.9	2.38	2.14	0.03	0.01	N/A	1.58	0.81	39.7	4.0	2.0	21.1	87.3	72.5	7.59	5.28	1.17	1.13	2.13	1.84

Abbreviations: IA, immunoadsorption; Ig, immunoglobulin.

**Figure 1:**
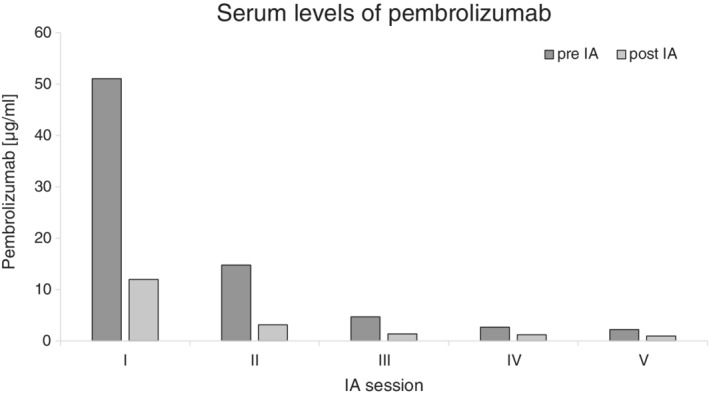
Pembrolizumab serum‐concentrations before (dark gray) and after (light gray) each immunoadsorption session using Ig omni columns

Serum samples were obtained before and after each IA session and kinetics of pembrolizumab levels were measured using an enzyme‐linked immunosorbent assay (ELISA) (Abcam, ab237652; see Figure 1).

After the first IA session, pembrolizumab serum‐concentration declined from 51.04 to 11.99 μg/mL (76.5% reduction from baseline). Between each session, pembrolizumab concentrations remained roughly stable. At the end of the treatment cycle, a pembrolizumab concentration of 0.96 μg/mL was measured, corresponding to a 98.1% decline from baseline.

## 3 DISCUSSION

In our patient, several sessions of TPE were performed successfully, to treat and prevent hyperviscosity‐related vision impairment. On progression, IA was chosen to reduce plasma IgM and fκLC. Rapid relief of hyperviscosity‐associated symptoms was accomplished. However, elimination of serum IgM, the pivotal villain of hyperviscosity, was less effective compared to prior TPE sessions (51.6% and 78% IgM‐reduction after two sessions of IA vs TPE, respectively). The observed elimination‐discrepancy between IgM and IgG is not completely clear, as the used broad spectrum columns (Ig omni 5) should similarly eliminate IgM and IgG.^8^ One advantage of IA compared to other extracorporeal techniques is the high elimination‐specificity of pathogenic substances. In case of severe adverse events, therapeutic apheresis is effective in eliminating toxic biological‐drugs immediately.^9^ Severe immune‐related adverse events might be such an indication for checkpoint inhibitors such as pembrolizumab.^10^ In addition, knowledge of drug‐specific pharmacokinetics during IA is also crucial for therapeutic dosing/re‐dosing‐schemes. Pembrolizumab levels can be removed by 80% to 90% by one cycle of 5 to 10 sessions of IA^11^ and even higher rates are expected with broad spectrum Ig columns. The initially measured pembrolizumab level before initiating IA in our patient (51.04 μg/mL) corresponds with the estimated target range, whereas the measured serum‐level after IA session I (11.99 μg/mL) declined even below the 10th percentile of pooled trough‐level data.^12^ Biochemical and pharmacokinetic properties of pembrolizumab and IgG resemble each other. Our analysis confirms that periprocedural elimination of serum IgG follows a comparable kinetic with pembrolizumab and therefore might serve as a surrogate for other IgG antibodies. Given the drug's small distribution volume and the long elimination half‐time, re‐application of pembrolizumab and other IgG monoclonal antibodies might be essential to sustain adequate therapeutic efficacy. Of note, blood levels and biological effects do not correlate necessarily. Natalizumab, a monoclonal antibody targeting alpha4‐integrin, is used to treat multiple sclerosis. In the case of progressive multifocal leukencephalopathy, a serious treatment complication, TPE not only accelerates the drug‐clearance but also leads to a clinically important desaturation of the receptor.^9^ Conversely, sustained B cell depletion, a pharmacodynamical marker of rituximab, may be detected even months after the drug has been eliminated by TPE. However, undetectable rituximab‐levels were associated with a higher risk of B cell‐reconstitution and consecutively disease relapse.^13^ A lack of evidence exists concerning scheduled re‐dosing of such potent biological drugs after therapeutic apheresis, taking into account the potential risk of severe adverse effects deriving from an overdose.

## Supporting information


**Appendix** **S1:** Supporting information
