# Supplementary material for: Lessons learned from immunoadsorption for hyperviscosity in IgM multiple myeloma—A case report
Source: J Clin Apher. 2020 Mar 6;35(3):227–30. doi: 10.1002/jca.21775 (PMC7383619; doi:10.1002/jca.21775)
Supplement: Supplementary file 1 — Appendix S1: Supporting information [file JCA-35-227-s001.docx]

**Supplementary appendix**

Supplementary table 1. Exemplary overview of different parameters before and after the initial two therapeutic plasma exchange sessions performed at first diagnosis of hyperviscosity syndrome.

| TPE  session # | Albumin  [3.56-4.61 g/dL] | | IgG  [0.630-1.610 g/dL] | | | Free light chain Kappa  [0.0033-0.0194 g/L] | | | IgM  [0.041-0.283 g/dL] | | | Complement C3  [90-180 mg/dL] | | Total protein  [6.7-8.6 g/dL] | | Ionized calcium [mmol/L] | | Total calcium  [2.20-2.55 mmol/L] | |
| --- | --- | --- | --- | --- | --- | --- | --- | --- | --- | --- | --- | --- | --- | --- | --- | --- | --- | --- | --- |
|  | pre (I) | post (II) | pre (I) | post (II) | % from baseline | pre (I) | post (II) | % from baseline | pre (I) | post (II) | % from baseline | pre (I) | post (II) | pre (I) | post (II) | pre (I) | post (II) | pre (I) | post (II) |
| I | N/A | N/A | 0.05 | 0.20 | **400.0** | 1.82 | 1.94 | **106.6** | 9.1 | 2.0 | **22.0** | N/A | N/A | 11.88 | 6.89 | 1.22 | 1.00 | 2.16 | 2.28 |
| II |  |  |  |  |  |  |  |  |  |  |  |  |  |  |  |  |  |  |  |

TPE, therapeutic plasma exchange; Ig, immunoglobulin

Supplementary table 2. Protein elimination rates during immunoadsorption.

| IA session # | Albumin [g/dL] | | | Fibrinogen [g/dL] | | | Estimated protein loss due to plasma loss [%] | IgG  [g/dL] | | | Free light chain Kappa [g/L] | | | IgM  [g/dL] | | | Total protein  [g/dL] | | |
| --- | --- | --- | --- | --- | --- | --- | --- | --- | --- | --- | --- | --- | --- | --- | --- | --- | --- | --- | --- |
|  | pre | post | % reduction | pre | post | % reduction | (albumin+fibrinogen)/2 | pre | post | % reduction | pre | post | % reduction | pre | post | % reduction | pre | post | % reduction |
| I | 2.04 | 1.93 | **-5.4** | 0.16 | 0.15 | **-6.3** | **-5.9** | 0.21 | 0.06 | **-71.4** | 2.04 | 1.36 | **-33.3** | 9.5 | 7.0 | **-26.3** | 11.45 | 9.18 | **-19.8** |
| II | 2.28 | 2.04 | **-10.5** | 0.14 | 0.14 | **0** | **-5.3** | 0.1 | 0.02 | **-80.0** | 1.98 | 1.15 | **-41.9** | 7.2 | 4.6 | **-36.1** | 10.17 | 7.44 | **-26.8** |
| III | 2.28 | 1.94 | **-14.9** | 0.18 | 0.15 | **-16.7** | **-15.8** | 0.05 | 0.01 | **-80.0** | 2.06 | 1.05 | **-49.0** | 5.9 | 3.6 | **-39.0** | 8.94 | 6.44 | **-28.0** |
| IV | 2.36 | 2.19 | **-7.2** | 0.25 | 0.21 | **-16.0** | **-11.6** | 0.04 | 0.01 | **-75.0** | 2.07 | 1.12 | **-45.9** | 4.7 | 2.8 | **-40.4** | 7.84 | 6.47 | **-17.5** |
| V | 2.38 | 2.14 | **-10.1** | 0.22 | 0.16 | **-27.3** | **-18.7** | 0.03 | 0.01 | **-66.7** | 1.58 | 0.81 | **-48.7** | 4.0 | 2.0 | **-50.0** | 7.59 | 5.28 | **-30.4** |
| **Average reduction per session [%]** |  |  | **-9.6** |  |  | **-13.3** | **-11.5** |  |  | **-74.6** |  |  | **-43.8** |  |  | **-38.4** |  |  | **-24.5** |

IA, immunoadsorption; Ig, immunoglobulin

**Material and methods:**

General remarks:

- Citrate anticoagulation led to an increase in total calcium (> 3 mmol/l) during the first two sessions. Thereafter, change to heparin as anticoagulation had no influence on total calcium values.
- Pembrolizumab concentrations were measured with “ab237652 Pembrolizumab ELISA Kit (Keytruda®)“, according to the provided manual (Version 1a last updated 17 January 2019). Owing to the methodology, concentrations were measured using a dilution of 1:100 (see manual paragraph 11.1/1.: “*Dilute samples at 1:100 (5 μL serum/plasma + 495 μL Assay buffer*”). Therefore, the pembrolizumab serum-concentrations provided here correspond to the measured values multiplied by factor 100.

Supplementary table 3. Immnoadsorption technical details

| Device | Column | Manufacturer | IA # | Anticoagulant (AC) | AC-ratio | Treated plasma volume [mL] | Patient’s calculated plasma volume* [mL] | Number of plasma volumes (relative) | Loading cycles per session | Frequency of procedures |
| --- | --- | --- | --- | --- | --- | --- | --- | --- | --- | --- |
| Life 21 | Ig omni® 5 | Miltenyi Biotech (Bergisch Gladbach, Germany) | I | ACD-A | 1:20 | 6474 | 2845 | 2.28 | 25 | Daily |
|  |  |  | II | ACD-A | 1:17 | 7198 | 2693 | 2.67 |  |  |
|  |  |  | III | Heparin | N/A | 7690 | 2600 | 2.96 |  |  |
|  |  |  | IV | Heparin | N/A | 5900 | 2697 | 2.19 |  |  |
|  |  |  | V | Heparin | N/A | 7700 | 2651 | 2.90 |  |  |

AC, anticoagulant; IA, immunoadsorption; ACD-A, Anticoagulant Citrate Dextrose Solution, Solution A

*Plasma volumes were calculated using Nadler’s formula for total blood volume for females.

Special remarks:

- Ig omni® 5 adsorber specifically removes immunoglobulins IgG (subclasses IgG1-IgG4), IgM, IgA, IgE, and immune complexes and free kappa and lambda light chains. Columuns contain recombinant antibody fragments as ligands which allow specific removal of over 70% of circulating IgG in each of five consecutive IA sessions, according to the manufacturer’s instructions.

Supplementary table 4. Therapeutic plasma exchange technical details

| Device | Version / Model | Manufacturer | Anticoagulant (AC) | AC-ratio | Treated plasma volume [mL] | Patient’s calculated plasma volume* [mL] | Number of plasma volumes (relative) | Replacement fluid | Number and frequency of procedures |
| --- | --- | --- | --- | --- | --- | --- | --- | --- | --- |
| Spectra Optia® | 61000 | TerumoBCT (Lakewood, CO, USA) | ACD-A | 1:16 | 3847 | 2999 | 1.28 | 5% human albumin (3500 mL) | First two sessions: daily |

AC, anticoagulant; ACD-A, Anticoagulant Citrate Dextrose Solution, Solution A

*Plasma volumes were calculated using Nadler’s formula for total blood volume for females.
